# Integrated functional neuronal network analysis of 3D silk-collagen scaffold-based mouse cortical culture

**DOI:** 10.1016/j.xpro.2020.100292

**Published:** 2021-01-25

**Authors:** Yu-Ting L. Dingle, Mattia Bonzanni, Volha Liaudanskaya, Thomas J.F. Nieland, David L. Kaplan

**Affiliations:** 1 Department of Biomedical Engineering, Tufts University, Medford, MA 02155, USA

**Keywords:** Cell culture, Microscopy, Neuroscience, Tissue engineering

## Abstract

Bioengineered 3D tunable neuronal constructs are a versatile platform for studying neuronal network functions, offering numerous advantages over existing technologies and providing for the discovery of new biological insights. Functional neural networks can be evaluated using calcium imaging and quantitatively described using network science. This protocol includes instructions for fabricating protein-based composite scaffolds, 3D *in vitro* culture of embryonic mouse cortical neurons, virally induced expression of GCaMP6f, wide-field calcium imaging, and computational analysis with open-source software and custom MATLAB code.

For complete details on the use and execution of this protocol, please refer to Dingle et al. (2020).

## Highlights

• Customizable 3D scaffold system to recapitulate *in vitro* neuronal networks
• Optical mesoscale detection of neuronal activity
• Structural, molecular, and functional characterization of the 3D *in vitro* system
• Network science approach reveals millimeter-scale topologies

## Graphical Abstract

**Figure:**
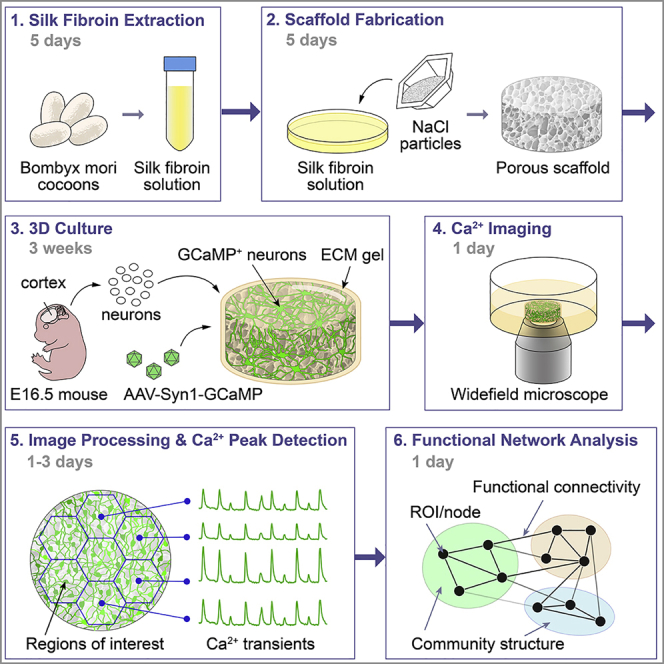

## Before you begin

### Extraction of silk fibroin fibers

**Timing: 3 h**

Silk fibroin is a natural polymer and a suitable material for both *in vivo* and *in vitro* biomedical applications because of its biocompatibility, tunable chemical and mechanical properties, and long-term stability *in vitro* yet with 100% degradability over time *in vivo* (Rockwood et al., 2011; Rouleau et al., 2020). We have previously reported several *in vitro* brain models, with both primary rodent neurons and human induced pluripotent stem cells (iPSCs), using porous scaffolds fabricated with silk fibroin and extracellular matrix composites. Such models recapitulated many key features of the brain tissue (Cairns et al., 2020; Cantley et al., 2018; Dingle et al., 2020; Liaudanskaya et al., 2020; Rouleau et al., 2020; Tang-Schomer et al., 2014).

**Figure 1:**
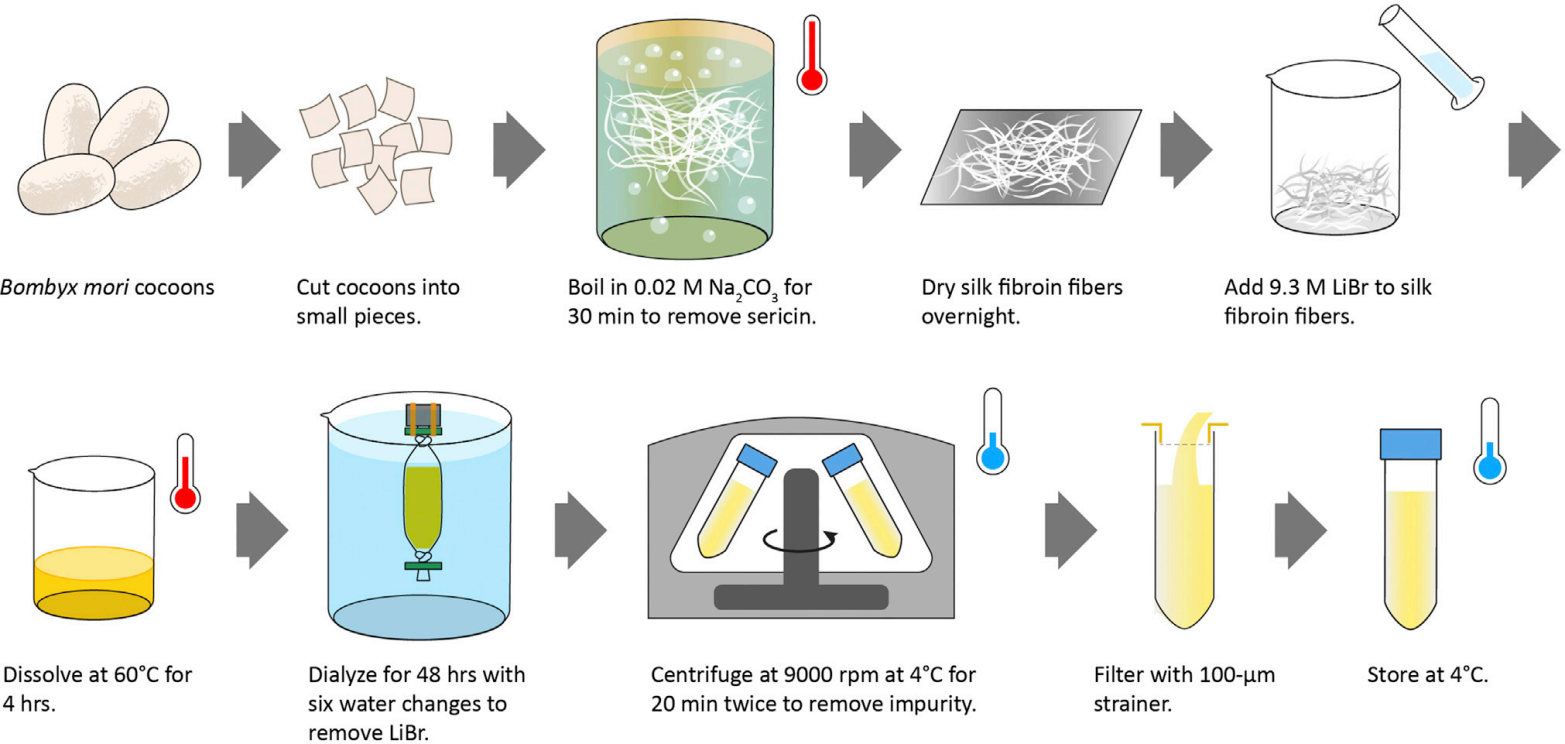
Preparation of silk fibroin solution See also Methods Videos S1, S2, S3, S4, and S5.

***Note:*** Approximately 15 whole cocoons are needed for a batch of 6.25 g cut cocoons.

Native *Bombyx mori* (silkworm) silk is composed of the core silk fibroin protein and the adhesive sericin proteins. This section describes the step-by-step instructions on the removal (degumming) of sericin and the extraction of fibroin fibers. See Figure 1.

***Note:*** Each batch of 6.25 g cut cocoons yield approximately 1.5 pieces of 10 cm diameter × 3–4 mm height silk sponge scaffold.

1. Cut *Bombyx mori* cocoons open. Dispose pupa and cut cocoons into small pieces that are no larger than 1.5 cm in any dimension. Remove any inner layers that are stained brown from the silkworm pupa. See Methods Video S1.

2. Weigh 6.25 g of cut cocoon pieces.
3. Boil 2.5 L research grade (RG) or deionized (DI) water in a 3 L metal beaker on a hot plate. Boil one extra beaker of RG or DI water for refilling evaporative water loss during boiling.
4. Add 5.3 g of Na_2_CO_3_ slowly to the boiling water (final concentration = 0.02 M).
5. Boil cocoon pieces for 30 min to degum the cocoons, which removes the sericin component from the silk fibroin fibers. Use serological pipettes to pull the fibers apart every 3–5 min while boiling. See Methods Video S2.

Methods Video S1. Bombyx mori cocoons are cut open and pupas are removed (extraction of silk fibroin fibers step 1) See also Figure 1.

***Note:*** Glass beakers are used in the video for better visualization.

6. Transfer degummed silk fibroin fibers to 4 L of cold RG or DI water in a plastic beaker. See Methods Video S3.

Methods Video S2. Degumming cocoons by boiling Cut cocoon pieces are placed in boiling 0.02 M Na2CO3 solution. Serological pipettes are used to periodically submerge and pull apart silk fibroin fibers. (Extraction of Silk Fibroin Fibers step 5). See also Figure 1.

7. Wash by manually pulling the fibers apart in the water followed by squeezing to dry. See Methods Video S4. Repeat this process 3–5 times.

Methods Video S3. Cooling silk fibroin fibers Degummed silk fibroin fibers are transferred to cold water at the end of 30-min boiling (Extraction of Silk Fibroin Fibers step 6). See also Figure 1.

8. Place the squeezed-dry silk fibroin fibers on aluminum foil and allow to dry completely at 20°C–25°C at places with high air flow, such as the fume hood.

***Note:*** A minimum of 12 h in the fume hood is usually sufficient.

**CRITICAL:** Dry silk fibers should feel stiff and crisp. If the silk fibers feel soft, continue drying process. Fibers that contain water will affect the solubilization in the subsequent step.

**Pause point:** Dry silk fibroin fibers can be stored at 20°C–25°C for at least a year.

Methods Video S4. Washing silk fibroin fibers Silk fibroin fibers are washed by manually pulling apart the fibers in water followed by squeezing to dry (Extraction of Silk Fibroin Fibers step 7). See also Figure 1.

### Solubilization of silk fibroin

**Timing: 3–4 days**

Silk fibroin is a natural polymer rich in hydrophobic β-sheet-forming blocks linked by small hydrophilic linkers (Rockwood et al., 2011). The degummed silk fibroin fibers extracted from the previous step retain their largely β-sheet secondary protein structure and thus remain water-insoluble. This section describes the use of lithium bromide (LiBr) to denature the β-sheets to create water-based fibroin solution. See Figure 1.

9. Weigh dry silk fibroin fibers.

**CRITICAL:** Silk fibers must be completely dry.

***Note:*** 6.25 g cocoons typically yield around 4 g dry silk fibroin fibers.

10. Prepare 9.3 M LiBr solution.
  **CRITICAL:** Stock LiBr should be dry and not clumpy. Keep the LiBr container closed and sealed using parafilm as much as possible to prevent LiBr from absorbing moisture from room air.
  a. Total LiBr solution (mL) needed = 4 × silk fibroin weight (g)
  b. Amount of LiBr (g) = (86.85 g/mol) × (9.3 mol/L) × (vol of total solution in step 10a) × (1 L/1000 mL)
  c. Starting volume of RG or DI water = 0.7 × volume from step 10a.
  d. Add RG or DI water from step 10c to a beaker. Slowly add LiBr to dissolve.
  e. Measure volume from step 10d with a graduated cylinder and bring to the final volume (calculated in step 10a) with RG or DI water.

11. Place silk fibroin fibers in a 100–200 mL glass beaker. Pour 9.3 M LiBr solution onto the silk fibroin fibers. Use a serological pipette to move fibers around to maximize exposure to LiBr solution. See Methods Video S5.

***Note:*** It is normal that the volume of LiBr solution appears very low in comparison to the amount of fibers.

12. Cover the beaker with aluminum foil and place in a 60°C oven for 4 h.

***Note:*** See Troubleshooting 1.

13. Prepare 4 L RG or DI water in a plastic beaker. Cut 20 cm of dialysis tubing (MWCO = 3,500 Da) and wet with RG or DI water. Tie and clip one end.
14. Transfer dissolved silk fibroin into dialysis tubing using a serological pipette.

***Note:*** Dissolved silk fibroin solution should appear slightly yellow in color and transparent. It should be viscous but not too viscous to pipette.

15. Leave approximately an additional 1/3 of tubing length, while minimizing the amount of air inside the tubing, for volume expansion of the silk fibroin solution during dialysis, as water will cross the dialysis membrane into the tubing. Remove air by squeezing and tie the open end. Clip both ends and secure to a float buoy with rubber bands.
16. Dialyze to remove LiBr by changing 4 L RG or DI water six times in the next 48 h on a stir plate. First two changes should be 1–2 h apart. Remaining four changes should be at least 4 h apart. Check conductivity of the final water with a handheld conductivity meter (see Key resources table), which should match fresh RG or DI water.
17. Collect silk fibroin solution in 50 mL conical tubes (does not need to be sterile).
18. Centrifuge silk fibroin solution at 4°C at 12,700 × *g* for 20 min. Pour silk fibroin solution into new conical tubes. Repeat centrifugation and pass silk fibroin solution through a 100-μm strainer into new conical tubes.
19. Measure concentration by drying 500 μL of silk fibroin solution in a weigh boat (smaller boats with diameter < 4 cm typically work better) in a 60°C oven for a minimum of 8 h.
  a. Silk fibroin concentration % (w/v) = 100 × dry weight of silk fibroin (g) ÷ 0.5 mL.

***Note:*** If the silk concentration is <6% (w/v), place silk fibroin solution back in new dialysis tubing and hang it in a fume hood for 8–12 h while exposed to air to allow evaporation.

20. Dilute silk fibroin solution with RG or DI water to 6% (w/v).

**Pause point:** This solution can be used immediately or stored at 4°C for up to 2 weeks.

***Note:*** See Troubleshooting 2.

Methods Video S5. Dissolving silk fibroin fibers Dry silk fibroin fibers are placed in a glass beaker and LiBr solution (9.3 M) is poured onto the fibers. The fibers are dispersed in the solution using a serological pipette to maximize exposure to LiBr (Solubilization of Silk Fibroin step 11). See also Figure 1.

### Preparation of silk scaffolds

**Timing: 5 days**

**Figure 2:**
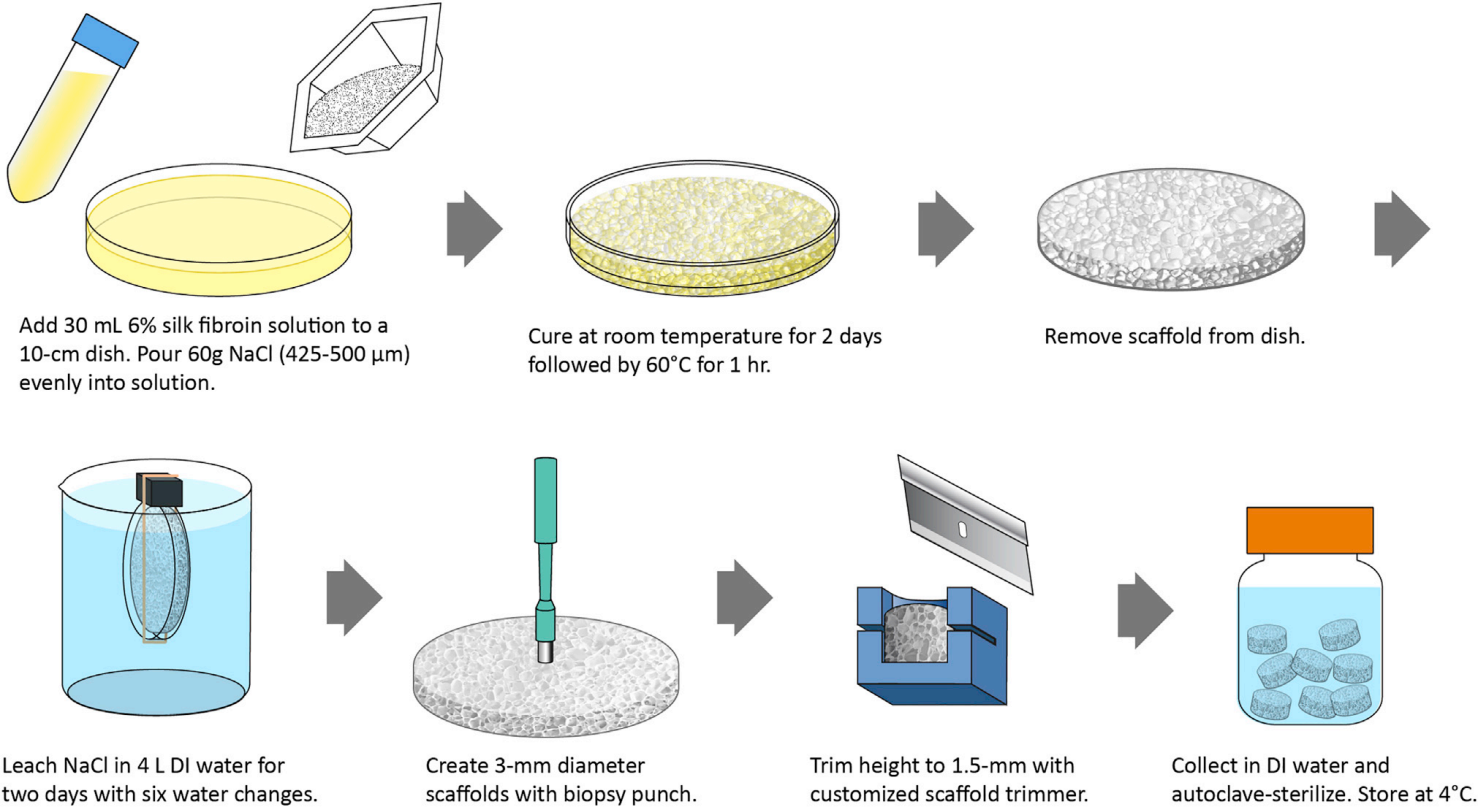
Preparation of silk scaffolds See also Methods Videos S6, S7, S8, and S9.

This section provides step-by-step instructions for fabricating porous silk fibroin scaffolds with pore sizes of 425–500 μm. These scaffolds will be used for 3D cortical culture. We had previously determined that 6% (w/v, in water) silk fibroin solution resulted in scaffolds with desirable mechanical properties for neuronal culture (Tang-Schomer et al., 2014). To initiate the self-assembly of aqueous silk fibroin into a scaffold composed of insoluble β-sheet crystalline structure, sodium chloride (NaCl) particles are incorporated in the silk solution, incubated, and later washed out to create pores. The resulting sponge material is manually trimmed to the final dimensions. See Figure 2.

***Note:*** Each 10 cm diameter × 3–4 mm height sponge requires 30 mL 6% (w/v) silk fibroin solution and 60 g sieved NaCl and yields at least 300 3-mm diameter scaffolds.

21. Stack stainless steel sieves in the top to bottom order of lid, 500-μm pore size sieve, 425-μm pore size sieve, and receiver tray (see Key resources table). Pour a small amount of NaCl at a time and sieve NaCl particles to size range of 425–500 μm. Shake until very little NaCl passes through the bottom 425-μm sieve. See Methods Video S6.

22. Prepare 60 g sieved NaCl particles in a large weigh boat (e.g., Fisherbrand 02-202-102, 110-mm diameter).
23. In a 10 cm plastic petri dish, first add 30 mL 6% (w/v) silk solution, and then slowly and evenly pour 60 g NaCl from the large weigh boat into silk solution. See Methods Video S7.

Methods Video S6. Sieving salt particles Sieves are stacked in the following order (top to bottom): lid, 500-μm sieve, 425-μm sieve, and receiver tray. A small amount of sodium chloride (NaCl) is poured on the 500-μm sieve. The stack is manually shaken to separate NaCl particle sizes of >500 μm (in the top 500-μm sieve). 425–500 μm (in the middle 425-μm sieve), and <425 μm (in the bottom receiver tray) (Preparation of Silk Scaffolds step 21). See also Figure 2.

**CRITICAL:** Achieving homogenous sponge formation, even distribution of NaCl particles is important. Slowly pour NaCl in a back and forth motion to add thin layers and rotate the dish after each layer.

24. Place the cover on the petri dish and place in a fume hood for 2 days, followed by placing in 60°C oven for 1 h. This allows silk fibroin to form water-insoluble β-sheet crystalline structure.
25. Fill a 4 L beaker with RG or DI water and rinse the cured sponge. Carefully break the edge of the plastic petri dish and slide a microspatula underneath the sponge to release the sponge from the dish. Soak and gently squeeze the sponge with DI water multiple times until the sponge is soft. See Methods Video S8.

Methods Video S7. Fabricating porous silk scaffolds In a 10-cm dish, first add 30 mL 6% (w/v) silk fibroin solution. Then add 60 g of NaCl particles from a large weigh boat by slowly pouring the salt into the solution in a back and forth motion to add thin layers. The dish is rotated after each layer (Preparation of Silk Scaffolds step 23). See also Figure 2.

26. Place the sponge in a clean petri dish lid and secure it to a float buoy using rubber bands. Wash the sponge in 4 L RG or DI water on a stir plate and change water every few hours over 1–2 days. Check conductivity of the final water, which should match fresh RG or DI water.

**Pause point:** The sponge can be stored in water in a petri dish, parafilm-sealed, at 4°C for up to 3 months.

***Note:*** See Troubleshooting 3.

27. Punch out 3-mm diameter cylindrical scaffolds with a biopsy punch. See Methods Video S9.

Methods Video S8. Releasing and leaching cured silk fibroin sponge from the 10-cm dish The dish is carefully broken manually, and the cured silk fibroin sponge (containing NaCl particles) is separated from the plastic with a microspatula. To first remove the majority of NaCl particles, the sponge is soaked and squeezed in 4 L water until it is soft (Preparation of Silk Scaffolds step 25). See also Figure 2.

**CRITICAL:** Always keep sponges wet in RG or DI water as dry sponges are fragile. See Troubleshooting 4.

28. Trim scaffold height to 1.5 mm with a razor blade. A useful method to keep height consistent is a customized, 3D printed scaffold trimmer. The SDL file of the scaffold trimmer can be downloaded from https://github.com/mattiabonzanni/Integrated-Functional-Neuronal-Network-Analysis-of-3D-Silk-Collagen-Scaffold-based-Cortical-Culture.
29. Place the scaffolds in an autoclavable container filled with RG or DI water. Autoclave-sterilize the scaffolds using a 120°C, 20-min liquid cycle.

**Pause point:** Store the sterile scaffolds at 4°C for up to 3 months.

Methods Video S9. Trimming scaffolds The salt-leached sponge is cut into 3-mm diameter cylindrical scaffolds using a biopsy punch (Preparation of Silk Scaffolds step 27). See also Figure 2.

## Key resources table

**Table:** 

REAGENT or RESOURCE	SOURCE	IDENTIFIER
**Bacterial and virus strains**		

AAV-hSyn1-GCaMP6f-P2A-nls-dTomato	Addgene	51085-AAV1 https://www.addgene.org/51085/
pENN.AAV.hSyn.TurboRFP.WPRE.RBG	Addgene	105552-AAV1 https://www.addgene.org/105552/

**Chemicals, peptides, and recombinant proteins**		

Sodium carbonate (Na_2_CO_3_)	MilliporeSigma	57795
Lithium bromide (LiBr)	MilliporeSigma	213225
Sodium chloride (NaCl)	MilliporeSigma	S7653
Poly-D-lysine (PDL)	MilliporeSigma	P6407
Laminin (0.5 mg/mL)	MilliporeSigma	MFCD00081739
Collagen type I solution	Corning	354236
Sodium hydroxide (NaOH)	MilliporeSigma	S2770
Calcein-AM	Thermo Fisher	C3100MP
Picrotoxin	Abcam	ab120315
Bicuculline	Abcam	ab120107
2,3-Dioxo-6-nitro-7-sulfamoyl-benzo[f]quinoxaline (NBQX)	Tocris	1044
(2R)-Amino-5-phosphonopentanoate (AP5)	MilliporeSigma	A8054
Tetrodotoxin citrate	Abcam	ab120055
β-Mercaptoethanol	MilliporeSigma	M3148
Dimethyl sulfoxide	MilliporeSigma	D2650
0.25% Trypsin-EDTA	Thermo Fisher	25200
DNase	MilliporeSigma	10104159001
Hank's balanced salt solution (HBSS)	Thermo Fisher	14025
Phosphate-buffered saline (PBS), sterile	Thermo Fisher	10010
Neurobasal medium minus phenol red	Thermo Fisher	12348
B27 supplement	Thermo Fisher	15240
Antibiotic-antimycotic	Thermo Fisher	15240
GlutaMax	Thermo Fisher	35050
Fetal bovine serum (FBS)	Thermo Fisher	10437
10× PBS	Fisher Scientific	BP399-4

**Oligonucleotides**		

Gabra1 (Taqman Assay)	Thermo Fisher	Mm00439046_m1
Gad2 (Taqman Assay)	Thermo Fisher	Mm00484623_m1
Gria1 (Taqman Assay)	Thermo Fisher	Mm00433753_m1
Grin1 (Taqman Assay)	Thermo Fisher	Mm00433790_m1
Pax6 (Taqman Assay)	Thermo Fisher	Mm00443081_m1
Rn18s (Taqman Assay)	Thermo Fisher	Mm03928990_g1
Shank3 (Taqman Assay)	Thermo Fisher	Mm00498775_m1
Slc17a7 (Taqman Assay)	Thermo Fisher	Mm00812886_m1
Slc1a3 (Taqman Assay)	Thermo Fisher	Mm00600697_m1
Syn1 (Taqman Assay)	Thermo Fisher	Mm00449772_m1
Dcx (Taqman Assay)	Thermo Fisher	Mm00438400_m1

**Software and algorithms**		

FluoroSNNAP	https://www.seas.upenn.edu/∼molneuro/software.html	n/a
Photoshop	Adobe	n/a
Illustrator	Adobe	n/a
NIS-Elements AR	Nikon	n/a
MATLAB	MathWorks	n/a
MATLAB Bioinformatics Toolbox Add-on	https://www.mathworks.com/products/bioinfo.html	n/a

**Deposited data**		

RunNetworkAnalysis MATLAB Code	https://github.com/mattiabonzanni/Integrated-Functional-Neuronal-Network-Analysis-of-3D-Silk-Collagen-Scaffold-based-Cortical-Culture	n/a

**Other**		

Weigh dishes (small, medium, large)	Fisher Scientific	02-202-100, 02-202-101, 02-202-102
Tissue culture dishes (35-mm, 60-mm, 100-mm)	Corning	430165, 430166, 430167
Syringe filters (0.22-μm, sterile)	MilliporeSigma	SLGV033RB
Syringes (5 mL)	Fisher Scientific	14-817-53
Cell strainers, 100-μm	Corning	431752
*Bombyx mori* cocoons	Tajima Shoji Co., Ltd, Japan	n/a
Dialysis tubing, molecular weight cutoff 3,500 Da	Fisher Scientific	21-152-9
Dialysis clips	Thermo Fisher	68011
Stainless steel spatula	Fisher Scientific	21-401-10
Biopsy punch, 3-mm diameter	Integra	12-460-406
Razor blades	Fisher Scientific	12-640
Scalpel blades	Fisher Scientific	NC9855170
Surgical scissors	Fine Science Tools	14060-09
Tissue forceps	Fine Science Tools	11052-10
Tissue culture plates (12-, 24-, 48-, 96-wells)	Fisher Scientific	08-772-29, 08-772-1, 08-772-1C, 08-7722c
Conical tubes (15 mL, 50 mL)	Fisher Scientific	14-959-53a, 14-432-22
Tissue culture plates, glass-bottom, 24-well	Cellvis	P24-1.5P
Nuclease-free 1.5 mL microcentrifuge tubes	Fisher Scientific	05-408-129
Homogenizer pestle	Fisher Scientific	12-141-364
Motorized tissue grinder	Fisher Scientific	12-1413-61
QIAshredder column	Qiagen	79656
Nuclease-free 0.2 mL PCR tubes or strips	Costar	07-200-681
Dumont #5 forceps	Fine Science Tools	11251-35 or11252-50
Angled forceps	Fine Science Tools	11251-35
3 L stainless steel beakers	Polar Ware	3W
4 L plastic beakers	Thermo Fisher	12014000
Float Buoys	Thermo Fisher	66432
Conductivity meter	Fisher Scientific	15-078-201
Stainless steel sieves (425 μm, 500 μm, lid/receiver)	Fisher Scientific	04-884-1AP, 04-884-1AN, 04-888A
Sterile, nuclease-free water	Thermo Fisher	10977015
Glass beaker (small)	Fisher Scientific	FB100100
Glass graduated cylinder	Fisher Scientific	08-550D
Dissection microscope	Nikon or equivalent	SMZ-754T or equivalent
Epifluorescence microscope equipped with a 4× objective, green fluorescence filter cube, and time-lapse acquisition function	Nikon or equivalent	Nikon Eclipse Ti-2 with Objective: CFI Plan Fluor DL 4× na 0.13 wd 16.5 mm Objective, filter #49002, and NIS-Elements AR software, or equivalent
Microscope stage-top incubator with temperature and CO_2_ controls	Bioscience Tools or equivalent	TC-MWP or equivalent
Confocal microscope with excitation laser and detector for red fluorescence	Leica or equivalent	SP8 or equivalent
NanoDrop UV-vis spectrophotometer	Thermo Fisher	2000 or equivalent
Thermocycler	Bio-Rad or equivalent	T100 or equivalent
qRT-PCR machine	Thermo Fisher	QuantStudio 5 or equivalent
3D Printer	Formlabs or equivalent	Form2 or equivalent ***Alternative:*** Order 3D printed parts from ShapeWays.com

**Critical commercial assays**		

Qiagen RNeasy Mini Kit	Qiagen	79656
iScript gDNA Clear cDNA Synthesis Kit	Bio-Rad	1725035
Taqman Gene Expression Master Mix	Thermo Fisher	4369016

**Experimental models: organisms/strains**		

Timed-pregnant (E16.5) C57BL/6 mouse	Charles River Laboratories	027

## Materials and equipment

**Table:** Stock solutions and aliquots preparation

Reagents	Stock solution concentration	Preparation procedures
Poly-D-lysine (PDL)	0.1 mg/mL	Dissolve 5 mg stock powder in 5 mL sterile water. Warm up the solution in 37°C bath, vortex, filter with a 0.22 μm syringe filter, and dilute with sterile water to a final concentration of 0.1 mg/mL. Store at 4°C for up to 1 month
DNase	10 mg/mL	Dissolve in water. Prepare 1 mL aliquots and store at −20°C for up to 1 year
Picrotoxin	100 mM	Add DMSO directly to stock vial. Prepare 4 μL aliquots and store at −20°C for up to 1 month
Bicuculline	20 mM	Add DMSO directly to stock vial. Prepare 4 μL aliquots and store at −20°C for up to 1 month
NBQX	10 mM	Add DMSO directly to stock vial. Prepare 4 μL aliquots and store at −20°C for up to 1 month
AP5	5 mM	Add water to stock vial. Transfer solution to conical tube and store at 4°C for up to 6 months
Tetrodotoxin	2 mM	Add water directly to stock vial. Prepare 20 μL aliquots and store at −20°C for up to 1 month
Laminin	(0.5 mg/mL)	Thaw stock vial on ice. Prepare 200–300 μL aliquots on ice and store at −20°C for up to 3 months
Adeno-associated virus (AAV)	N/A	Record titer information. Thaw stock vial on ice. Prepare 10–15 μL aliquots on ice and store at −80°C for up to 1 year

**CRITICAL:** Picrotoxin, bicuculline, NBQX, AP5, and TTX are neurotoxins. Prepare stock solutions in chemical hood with proper PPE and safety practice. Add solvent directly to the stock vials.

***Note:*** Prepare PDL, DNase, Laminin, and AAV with sterile techniques. Use standard biosafety practice when handling AAV.

**Table:** 

PDL/Laminin coating solution	Final concentration	Amount
PDL (0.1 mg/mL)	0.1 mg/mL	10 mL
Laminin stock (0.5 mg/mL)	10 μg/mL	200 μL
Total		10 mL

**CRITICAL:** Thaw laminin stock on ice or at 4°C. Laminin should never be thawed at 20°C–25°C or at 37°C. Make PDL/laminin coating solution fresh on the day of coating.

**Table:** 

Neuro medium	Final concentration	Amount
Neurobasal medium minus phenol red	n/a	480 mL
B27 supplement	2%	10 mL
Antibiotic-antimycotic	1%	5 mL
GlutaMax	2 mM	5 mL
**Total**	**n/a**	**500 mL**

***Note:*** Store at 4°C for up to 1 month.

**Table:** 

Neuro medium Plus 5% FBS	Final concentration	Amount
Neuro medium	n/a	38 mL
FBS	5%	2 mL
**Total**	**n/a**	**40 mL**

***Note:*** Store at 4°C for up to 2 weeks.

**Table:** 

Neuro medium Plus 10% FBS	Final concentration	Amount
Neuro medium	n/a	36 mL
FBS	10%	4 mL
**Total**	**n/a**	**40 mL**

***Note:*** Store at 4°C for up to 1 month.

**Table:** 

Trypsin/DNase	Final concentration	Amount
0.25% Trypsin-EDTA	n/a	12 mL
DNase	0.3 mg/mL	360 μL
**Total**	**n/a**	**12 mL**

***Note:*** Prepare 2 mL aliquots and store at −20°C for up to 6 months.

## Step-by-step methods

### Scaffold coating

**Timing: 1 h each on day −1 and day 0**

**Figure 3:**
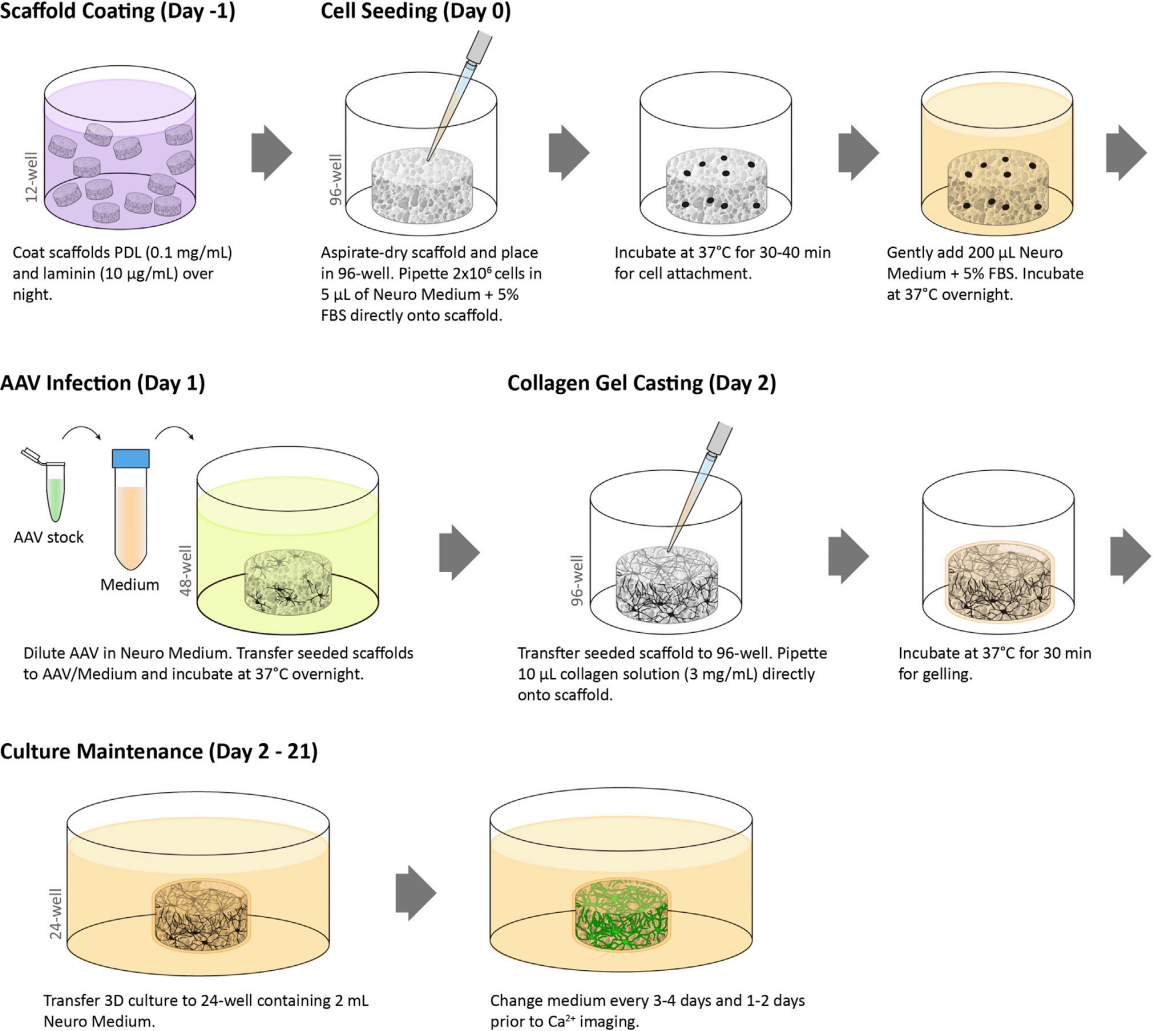
3D GCaMP^+^ neural culture setup

This step coats the silk scaffolds with PDL and laminin, which is essential for cell adhesion to the silk scaffolds. This procedure should be done in a biosafety hood under sterile conditions. See Figure 3.

1. Prepare fresh PDL/Laminin Coating Solution (See Materials and equipment).
2. Place up to 30 scaffolds per well in a 12-well plate and add 2 mL PDL/laminin coating solution per well.

***Optional:*** See additional steps in Quality Control #1 - 2D Culture (steps 84–89).

3. Incubate at 37°C for 12–24 h.
4. On the following day, aspirate the PDL/laminin coating solution from the scaffolds. Wash the scaffolds with PBS (4 mL/well) twice with 10 min incubation. Soak once with Neuro Medium at 37°C for at least 1 h, and soak with Neuro Medium Plus 5% FBS at 37°C for at least 1 h until cells are ready for seeding.

### Cortical neuron isolation and cell seeding

**Timing: 5–6 h on day 0**

This section describes the isolation of primary neural cells from mouse cerebral cortices and the subsequent seeding of cells onto 3D silk scaffolds. See Figure 3.

***Note:*** Proper institutional approval per animal protocols must be attained prior to embarking on this method.

***Note:*** Animal dissection should be done in a biosafety hood under sterile conditions. If the lab is not equipped with a dissection microscope inside a biosafety hood, it is acceptable to perform dissection on the bench. Tissue dissociation and cell culture should be done in a biosafety hood under sterile conditions.

***Optional:*** Refer to “Quality control assay #1, steps 84–89” and “Quality control assay #3, steps 94–97” to examine the viability of the neurons.

5. Chill 150–200 mL HBSS on ice.
6. Fill three 10 cm dishes with ice-cold HBSS. Fill several 60 mm dishes with ice-cold HBSS and keep on ice.
7. Sacrifice timed-pregnant (E16.5) mouse. Shave abdomen and clean with 70% ethanol. Hold abdomen with tissue forceps and cut open skin with surgical scissors.

***Note:*** To avoid contamination from the skin, avoid reusing these tools.

8. With separate scissors and forceps, remove and place the uterus in the first 10 cm dish of ice-cold HBSS to remove excess blood.

***Note:*** Blood will adversely affect the health of the cells.

9. Transfer the uterus to the second dish of HBSS, extract embryos, and place embryos in the third dish of HBSS.
10. Decapitate the embryos with scissors and place the heads in the third 60 mm dish of HBSS on ice.

***Note:*** Keep tissues on ice as much as possible to maintain viability.

11. Under a dissection microscope, stabilize the head by inserting angled forceps into the eye sockets and hold down. Gently make a midline cut with a scalpel blade and peel back the skin and skull with Dumont #5 forceps. Carefully remove the brain with a spatula and place the brain in ice-cold HBSS in a 60 mm dish.
12. Separate the cerebellum and the hemispheres with forceps.
13. Peel and remove the meninges with forceps.
14. With the medial side facing up, remove the midbrain and separate the striatum and cortex with forceps. Collect cortices in a 50 mL conical tube with HBSS on ice.
15. Remove HBSS from the conical tube containing cortices. Rinse the cortices with 30 mL PBS.
16. Carefully remove the PBS and add 2 mL of 0.25% Trypsin + 0.3 mg/mL DNase solution.
17. Transfer tissues/Trypsin/DNase to a 35-mm dish and incubate at 37°C for 20 min.
18. Add 2 mL Neuro Medium Plus 10% FBS to the tissue/Trypsin/DNase and transfer the 4 mL mixture to a 15 mL conical tube.
19. Add an additional 4 mL of Neuro Medium Plus 10% FBS. Triturate with a 5 mL pipette tip 20–25 times to mechanically dissociate the tissues.
20. Pass the solution through a 100-μm cell strainer into a new 50 mL conical tube. Rinse the strainer with an additional 4 mL Neuro Medium Plus 10% FBS.
21. Count the cells (recommended at 1:10 dilution) using Trypan Blue dye.

***Note:*** Expected viability >80% and 6–10 × 10^6^ live cells per pup.

22. Centrifuge at 210 × *g* for 5 min.
23. Remove supernatant.
24. The final seeding cell number and volume per scaffold is 2 × 10^6^ cells in 5 μL (i.e., 400 × 10^6^ cells/mL) of Neuro Medium Plus 5% PBS. Because the cell pellet accounts for a significant volume at this density, only add 3.5 μL per 2 × 10^6^ cells of Neuro Medium Plus 5% PBS to resuspend the cell pellet.

***Note:*** We have determined empirically that the cell pellet accounts for approximately 30% of the final volume.

25. Aspirate dry the PDL/laminin-coated scaffolds and transfer each scaffold to the center of a well in a 96-well plate.

**CRITICAL:** Do not dry the scaffolds until the cell suspension is ready for seeding and it is recommended to dry no more than 30 scaffolds at a time. Dry scaffolds quickly become fragile, so cell suspension needs to be added as soon as possible.

26. Resuspend cells by triturating 2–5 times. Avoid air bubbles.

**CRITICAL:** Shear force can affect cell viability. Once the suspension seems homogeneous, stop pipetting.

27. Add 5 μL of cell suspension directly onto the scaffold.

***Optional:*** A repeater pipette can be used to speed up the seeding process.

***Note:*** If scaffold gets stuck on the pipette tip, use another pipette tip or forceps to gently dislodge.

28. Incubate seeded scaffolds for 30 min at 37°C, 5% CO_2_, 95% relative humidity (RH) for initial cell attachment.
29. Gently add 200 μL Neuro Medium Plus 5% FBS to each well and incubate at 37°C, 5% CO_2_, 95% RH for 18–24 h.

### Adeno-associated virus (AAV) infection

**Timing: 1 h on day 1**

This section describes the protocol for infecting 3D cultures with AAV-Syn1-GCaMP6f (Chen et al., 2013). Cells adhere onto the 3D silk scaffold surfaces overnight before neurons are infected with AAV-Syn1-GCaMP6f, an adenoviral system for expressing the genetically encoded calcium indicator (GECI) under the human synapsin 1 promotor. This procedure should be done in a biosafety hood under sterile conditions. See Figure 3.

30. Warm up Neuro Medium, 500 μL per scaffold, to 37^o^C.
31. Thaw AAV1-hSyn1-GCaMP6f-P2A-nls-dTomato (referred as AAV hereafter) stock solution on ice.

***Optional:*** See additional steps in Quality Control #3 -Viability Staining (steps 94–97), Quality Control #4 – Visualizing Structural Network (steps 98–100), and Quality Control #5 – Gene Expression (steps 101–109).

32. Dilute AAV in pre-warmed Neuro Medium (serum-free) to a final concentration of 2 × 10^10^ GC/mL.
33. Add 500 μL AAV/Neuro Medium to individual wells of 48-well plate for infection (1 well for each scaffold to be infected).
34. Using forceps, transfer each scaffold into one of these wells containing AAV/Neuro Medium. Incubate at 37°C for 24 h.

***Note:*** It is normal to have many unadhered cells on the bottom of the 96-well plate from day 0. Seeding efficiency is estimated to be around 60%–70%.

### Collagen I gel casting and culture maintenance

**Timing: 2–3 h on day 2 and 30 min every 3–4 days**

This section describes casting of Collagen Type I onto the seeded 3D scaffolds. By this day, cells have adhered to the surfaces throughout the 3D scaffold. Collagen gel will fill the pores of the scaffold to provide an additional substrate for 3D neurite extension. This procedure should be done in a biosafety hood under sterile conditions. See Figure 3.

35. Warm up Neuro Medium, 2 mL per scaffold, to 37^o^C.

36. Prepare 3 mg/mL Collagen Type I solution by mixing Collagen stock solution, 1 M NaOH, 10× PBS, and cold media according to the following calculations. Keep solution on ice.
  **CRITICAL:** To account for pipetting loss, prepare at least 20 μL per scaffold and at least 500 μL total.
  ***Note:*** Use Collagen Type I stock solution with concentration >3.5 mg/mL if possible. Otherwise, there will not be enough room for 10× PBS and/or medium.
  a. Collagen Type l I stock volume = [final volume] × [final concentration 3 mg/mL] ÷ [stock concentration]
  b. NaOH = 0.023 × [Col I stock volume]
  c. 10× PBS = 0.1 × [final volume]
  d. Cold media = [final volume] − [Col I stock volume] − [NaOH] − [10× PBS]
  e. Check pH, which should be in the neutral range.

37. Transfer each scaffold to an individual well of a 96-well plate.

**CRITICAL:** Do not remove excess medium from the scaffold as it may result in cell detachment.

38. Add 10 μL Collagen Type I solution directly onto each scaffold.
39. Incubate at 37°C, 5% CO_2_, 95% RH for 30–40 min.

**CRITICAL:** Do not incubate longer as cell health is affected by the lack of medium.

40. In 24-well glass-bottom plates, add 2 mL Neuro Medium to each well and avoid edge wells. Fill empty wells and space between wells with PBS.

***Note:*** The PBS helps with maintaining humidity during the culture period and with maintaining temperature during Ca^2+^ imaging in the subsequent step.

41. Transfer the now complete 3D cultures to the 24-well glass-bottom plates (one scaffold/well).
42. Maintain cultures at 37°C, 5% CO_2_, 95% RH with 50% medium change every 3–4 days.
43. Change 50% medium 1–2 days prior to Ca^2+^ imaging.

**CRITICAL:** Avoid changing or adding new medium on the day of imaging because sudden change in medium composition may cause temporary alternation in baseline activities.

### Ca^2+^ imaging on a wide-field epifluorescence microscope

**Timing: variable (approximately 1 h preparation plus 10–30 min per sample)**

**Figure 4:**
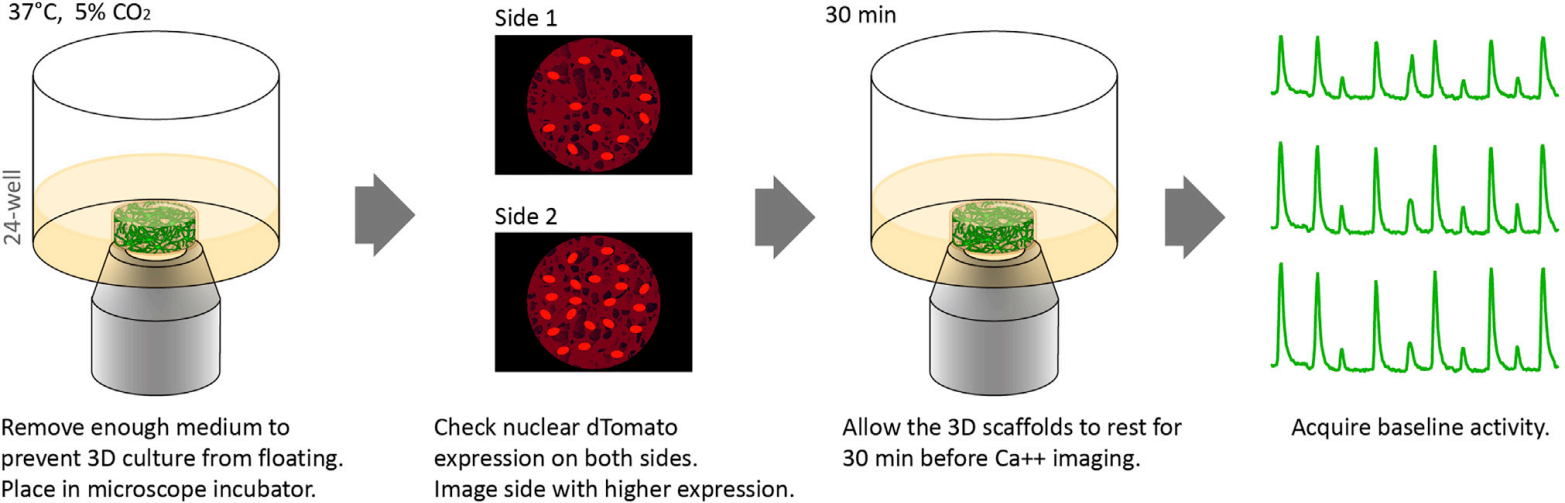
Wide-field Ca^2+^ imaging of 3D cultures See also Methods Video S10.

The functional measurement of the 3D system is achieved using the genetically encoded calcium indicator (GCaMP) to detect changes in the cytoplasmic Ca^2+^ concentrations in the neural culture (Chen et al., 2013). A large field of view is needed to investigate functional networks at mesoscale, i.e., activity of populations of neurons across hundreds of μm (Betzel and Bassett, 2017). This section describes time-lapse recordings of Ca^2+^ transients in neurons expressing GCaMP at a low magnification/large field of view to capture the entire 3D culture projection. The image data are used for network analysis in the subsequent sections. See Figure 4.

***Note:*** Ca^2+^ imaging is recommended at 3 weeks of culture. GCaMP expression is sufficient at 2 weeks of culture for Ca^2+^ imaging but higher sample-to-sample variability has been observed at an earlier time point.

***Note:*** For terminal time points, this procedure does not need to be done under sterile conditions. For longitudinal studies, conduct this procedure under sterile conditions as much as possible.

44. Set the microscope stage-top incubator to 37°C and 5% CO_2_.
45. Remove enough medium (approximately 1.5 mL per well) so that the 3D culture is not floating, but still submerged in medium.

**CRITICAL:** 3D cultures must not be moving during time-lapse imaging.

***Note:*** For longitudinal studies of the same set of 3D cultures, this medium should be removed under sterile conditions, saved and placed back into the original wells/plates after Ca^2+^ imaging is complete. Use sterile tools and tips during imaging for longitudinal study samples.

46. Place the plate in the microscope stage-top incubator.
47. Use the red fluorescence channel to visually check the density of dTomato-positive cells. Flip the 3D culture with forceps (or microspatula) to determine which side has the higher number of dTomato^+^ cells.

***Note:*** It is not unusual if one side has significantly more dTomato^+^ cells than the other.

48. Leave 3D cultures undisrupted in the microscope stage-top incubator for at least 30 min to reach steady state.
49. Choose the microscope objective based on the required field size and resolution. In this study, a 4× objective, with 4.15 mm × 3.51 mm field size was used to capture the full 3D culture projection.
50. Set the acquisition rate, time-lapse duration, FITC channel exposure time, gain, and binning. A minimum 1 Hz is required (higher frequency recommended) to capture Ca^2+^ events, and the acquisition parameters should be optimized according to user’s microscope and camera setup. Table 1 describes the factors to consider for each acquisition parameter.

**Table 1:** Time-lapse image acquisition setup for Ca^2+^ imaging

Image acquisition factors	Options/Explanations	Used in this study with Nikon Eclipse Ti-2 Microscope and Sola SE-Illuminator
Objective	• Low (4×) – larger field of view. Enables millimeter-sized network analysis. Lower or no cellular resolution • High (10× or higher) – smaller field of view. Higher and possible cellular resolution	4×
Fluorescence output	• Low – less photobleaching. Requires longer exposure • High – more photobleaching. Requires shorter exposure	Low (2%)
Gain	• Low gain – less noise. Requires longer exposure • High gain – more noise. Requires shorter exposure	High gain (gain-4)
Binning	• No binning – high resolution. Low signal and requires longer exposure • Binning – reduced resolution. Higher signal and requires shorter exposure	3 × 3 binning
Exposure	Must be short enough to achieve the required acquisition speed	50 ms
Speed (interval)	Must be ≥1 Hz to capture Ca^2+^ events. Higher frequency provides better resolution of the peaks	5 Hz (200 ms)
Duration	• Short – smaller file size and requires less computational power for analysis • Long – captures better activity patterns but larger file size	1–3 min
51. Move the stage so that the 3D culture is centered in the image field of view.
52. Record time-lapse series of green fluorescence signal.

***Note:*** If there is no GCaMP signal or change in fluorescence intensity, see Troubleshooting 5 and Troubleshooting 6.

***Optional:*** See additional steps in Quality Control #2 – Drug Response (steps 90–93).

53. Export the time-lapse files as OEM .tif image stacks.

### Generating region-of-interest (ROI) mask for network analysis

**Timing: <1 h**

**Figure 5:**
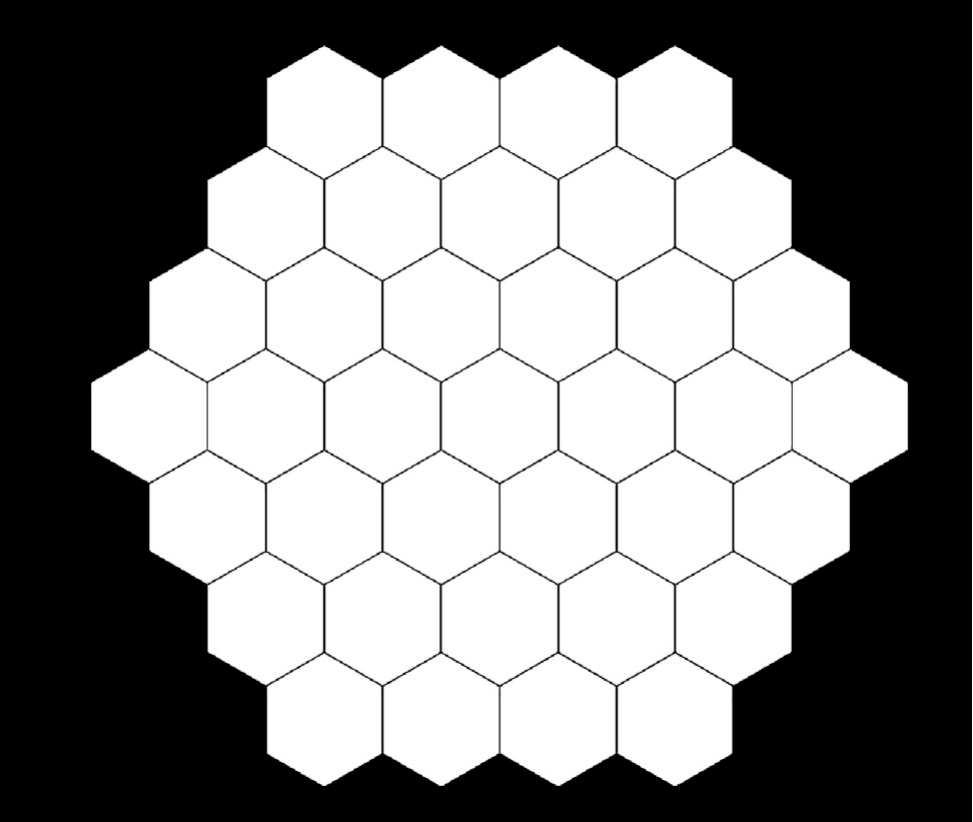
ROI masks composed of 37 500-μm center-to-center distanced hexagons

This section describes how to design and convert to binary mask using Adobe Illustrator and Adobe Photoshop. The goal is to create a mask containing 37 hexagonal ROIs, with 500 μm center-to-center distancing (Figure 5). The mask must be binary (black/white) in Bitmap format in order to be used in the subsequent Image Processing Step.

***Alternatives:*** Most microscope software has built-in ROI mask functions and can be utilized if it generates a mask in black/white binary and bitmap format. We also provide a pre-made mask with FOV size 4.15 mm × 3.51 mm (852 px × 720 px), which can be downloaded at https://github.com/mattiabonzanni/Integrated-Functional-Neuronal-Network-Analysis-of-3D-Silk-Collagen-Scaffold-based-Cortical-Culture.

54. Obtain μm-to-pixel scale information from the microscopy software.

**CRITICAL:** All the following steps are described in μm, but converted values (in pixels) should be used.

55. In Adobe Illustrator, *File → Create new → Custom →* enter the width and height (pixels) of the time-lapse image and in *Advanced Settings* *→* *Color Mode*, select RGB.
56. Double click on the Fill color and change it to black (R=0, G=0, B=0)
57. From the Tools window, select Rectangle Tool.
58. Drag and draw a black rectangle to completely cover the artboard.
59. Double click on the Fill Color and change it to white (R=255, G=255, B=255)
60. Switch to Polygon Tool (click and hold Rectangle Tool to open Polygon Tool). Single click and enter Radius and Sides (6). A white hexagon should appear.
61. Use Selection Tool to select the hexagon. Use Rotate Tool if necessary.
62. From the top menu, *Object → Transform → Move.* Enter Distance (500 μm) and Angle, Check Preview, and Copy.
63. Repeat Move/Copy with the correct angles until the mask pattern is generated.

***Note:*** Multiple selected hexagons can be grouped by *Right Click → Group*. The Move/Copy comment will apply to all the hexagons in the group.

64. *File → Export → Export As* and save as TIF, Color Mode: Grayscale, Anti-aliasing: None, Resolution: 72 dpi (LZW compression is ok).

65. Open the TIF file in Adobe Photoshop. Image *→* Mode, switch to Bitmap.

***Alternatives:*** Open the TIF file in NIS-elements. Select the white hexagons as ROI and save ROI as separate TIF.

66. Save as TIF.

***Note:*** LZW compression is fine. This generates a binary mask that can be read by FluoroSNNAP in the subsequent steps. An RGB or grayscale format mask is incompatible.

### Time-lapse image processing on FluoroSNNAP

**Timing: variable (typically 20–30 min per file)**

This section describes using open-source software (FluoroSNNAP) to extract fluorescence intensity *versus* time traces and identifying Ca^2+^ events in each ROI (Patel et al., 2015). A global synchronization index is calculated in this step. Cross-correlation of Ca^2+^ traces from each ROI pair is calculated and a matrix of cross-correlation coefficients of all the paired ROIs is generated.

67. Download and install FluoroSNNAP.
68. Run FluoroSNNAP as an administrator.

***Note:*** If FluoroSNNAP closes immediately, see Troubleshooting 7.

69. Select *File → Add folders containing .tif stacks.* Select the folder containing the stack files to analyze. After the path appears in the left window, click on the path.
70. Select the .tif stack file in the right window, select *Pre-Processing → Segmentation GUI → select a frame →#1.*
71. The Segmentation GUI window appears. Select *File → Open Segmentation.*In the File Type dropdown menu, select *All Files*. Select the ROI mask created from the previous section.
72. Select *File → Save segmentation.* A .tif file and a .mat (Microsoft Access Table Shortcut) file will be generated.

***Note:*** These files are subsequently used by the program for further analysis in the subsequent section.

73. Select *Analysis → Preferences → Analysis Modules* and leave only the following modules checked
  a. Convert raw fluorescence
  b. Detect onset of calcium event
  c. Perform synchronization analysis
  d. Estimate functional connectivity
74. *Select Analysis → Preferences →*
  a. Acquisition frame rate: Enter the acquisition rate used for the recording, i.e., 5 Hz
  b. Baseline fluorescence: F_0_ = 10 s of previous and 20th percentile
  c. Ca^2+^ event detection: template-based, threshold = 0.85, and minimum dF/F amplitude = 0.01.
  d. Synchronization analysis: method = phase (1), number of times to perform surrogate resampling = 20, minimum size of synchronization cluster = 3
75. Select *Analysis → Preferences → Functional Connectivity.* In this study, we use Cross-Correlation for functional connectivity analysis. See Troubleshooting 8 for recommendations on the choices of the functional connectivity. Save preference.
76. Select *Analysis→ Process single file.* At the end of the analysis run, a *processed_analysis.mat* file is generated for each time-lapse and will be used for further analysis.

***Note:*** If FluoroSNNAP freezes, see Troubleshooting 9.

77. Rename the *processed_analysis.mat* file to *processed_analysis_(your_file_name).mat* file in the folder, but do not rename it in MATLAB (the *RunNetworkAnalysis.m* script described in the next section works under the assumption that the name of the file loaded in the Workspace is *processed_analysis.mat*).

**CRITICAL:** Rename the processed-analysis.mat file in the folder after it is generated, or it will get overwritten by FluoroSNNAP when the next time-lapse stack is processed.

***Optional:*** To check the output file is in the correct format, open the *processed_analysis_(your_file_name).mat* in MATLAB. It should load a 1 × 1 processed_analysis.mat structure, containing 21 fields. To view the cross-correlation coefficient matrix, open *FC→CC→C*.

### Network analysis with *RunNetworkAnalysis.m*

**Timing: variable (typically 1–2 min per file)**

This section describes a step-by-step walkthrough of using *RunNetworkAnalysis.m* which can be accessed at https://github.com/mattiabonzanni/Integrated-Functional-Neuronal-Network-Analysis-of-3D-Silk-Collagen-Scaffold-based-Cortical-Culture. This custom MATLAB code loads the *processed_analysis.mat* file generated from FluoroSNNAP in the previous step, extracts and organizes the variables and computes parameters that describe the network properties (described in detail after the steps). This code runs on MATLAB (version 2019 or later), and Bioinformatics Toolbox Add-on is required.

In brief, the analysis is based on graph-theory descriptors. A graph (network) is composed of nodes interconnected by edges (Bullmore and Sporns, 2009). In our analysis, each ROI is a node and the statistical dependence (assessed using the cross-correlation method in this manuscript) between two ROI activities is an edge. The computed parameters describe several features of the reconstructed network topology.

The protocol here focuses on mesoscale activity, namely the statistical dependence of the activities of clusters of neurons, to capture the population, rather than single-cell activities, of the entire 3D culture. The user can however customize the size and shape of the ROIs to address different questions. It follows that different spatial parcellations lead to different interpretations of the resulting network (Messé, 2020).

***Note:* Descriptions of Network Parameters - Node.** Each ROI is represented in the network as a node. Overall, a node can be defined from a single neuron to entire circuits; considering the multiscale organization of neuronal networks, there is no single, privileged scale (Hallquist and Hillary, 2019).

***Note:* Descriptions of Network Parameters - Edge**. The edge between two nodes represents their functional connectivity, e.g., the statistical dependence between their activities. The value of functional connectivity between two nodes must not be necessarily interpreted as an indication of a physical connection (structural connectivity).

***Note:* Descriptions of Network Parameters - Binary versus Weighted networks**. A weighted network is a network whose edges have values between 0 and 1. An edge weight represents the weighted statistically dependence of the activity of the two nodes. On the other hand, such estimates are associated with measurement noise (small nonzero and/or negative weights). A binary network could be thus derived through a cutoff procedure with a user-defined threshold to the edge weights. An edge is preserved if its weight is higher than the threshold and removed otherwise. The preserved edges assume a value of 1 in the matrix; the eliminated edges assume a value of 0 instead.

78. Download and unzip Matlab Code-Integrated Functional Neuronal Network Analysis of 3D Silk-Collagen Scaffold-based Cortical.zip. This folder contains the following files:
  a. avg_clus_matrix.m
  b. avg_path_matrix.m
  c. circularGraph.m
  d. clustering_coef_matrix.m
  e. matrixExtraction.m
  f. NetworkAnalysis.m
  g. node.m
  h. processed_analysis_sample.mat
  i. RunNetworkAnalysis.m
  j. singleROIAnalyis.m
79. In MATLAB, change directory to the *RunNetworkAnalysis.m* folder.
80. Run *RunNetworkAnalysis.m* file.

***Note:*** See Troubleshooting 10.

81. When prompted, select the *processed_analysis_(your_file_name).mat* of interest.

***Optional:*** Run the sample test file *processed_analysis_sample.mat* to ensure the program operates smoothly.

82. The code will ask:
  “Do you want to binarize the matrix?”
  The user can either run the weighted or binary matrix.
    a. Weighted graph analysis: Select No.
    b. Binary graph analysis: Select Yes and enter the threshold value.
    c. Save file as: Enter a file name for the exported excel spreadsheet containing all outputs.

***Note:*** This threshold value is user-defined. See Troubleshooting 11. To view the raw cross-correlation coefficient values, see Time-lapse Image Processing on FluoroSNNAP section, step 79 or the *Matrix.mat* output from the code.

**Figure 6:**
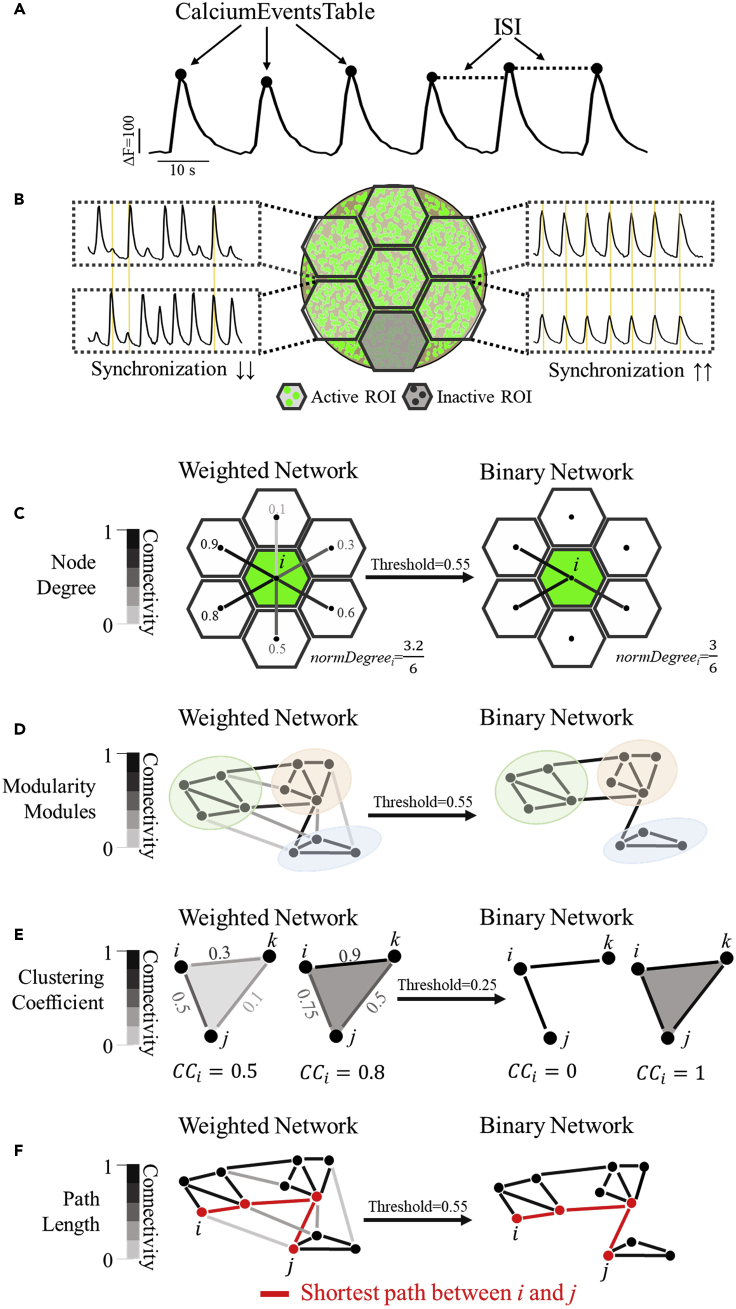
Summary of single node and network analysis outputs

***Note:*** In this analysis, each ROI is defined as a node, and the terms ROI and node therefore can be used interchangeably. See Troubleshooting 12 for error message regarding negative weighted edge.

***Note: CalciumEventsTable (Calcium Events Table)*** – A calcium event is a transient and synchronous change in fluorescence of a cluster of neurons in response to their intracellular elevation of Ca^2+^ associated with an action potential. In the Calcium Events Table, each column represents a ROI, and the data represents the time of peak (s) of the Ca^2+^ events per ROI (a peak is defined as the time at which a calcium event reaches its maximum value).

***Note: ISITable (Inter-spike Interval Table) -*** The inter-spike interval (ISI) is the average time (s) between two consecutive Ca^2+^ events (Figure 6A). It is computed for each ROI and organized in a tabular format. The ISI is calculated as:

83. The code outputs the following variables that can be viewed in MATLAB and in the exported Excel spreadsheet. These variables are summarized in Table 2 and Figure 6 and described in greater detail below:

**Table 2:** List of output parameters from *RunNetworkAnalysis.m* script and brief descriptions

Data	Variable name or location		Brief description
ROI/node-based	*CalciumEventsTable*		timing of calcium peaks in each ROI (s)
	*ISITable*		inter-spike intervals (ISI) per ROI (s)
	*SingleParameterTable*	*AverageFrequency*	average calcium event frequency (events/min) of the sample
		*PercentageActiveROI*	percentage of ROIs with at least one Ca^2+^ event
		*SyncIndex*	global synchronization index
	*NodeDegree*	*NodeIndex*	index of each ROI
		*Degree*	node degree of each ROI
Network-based	*FinalTableNetworkData*	*N*	number of nodes (ROIs)
		*Modularity*	modularity value
		*NumberModules*	number of modules
		*AverageEdgeWeight*	average edge weight
		*AverageClusteringCoef*	average clustering coefficient
		*AveragePathLength*	average path length
	*ModulesComposition*	*(First column)*	module index
		*(Second Column)*	number of ROIs in the module
		*(Third Column)*	index of the ROIs per module
	*Node’s degree distribution*		kernel density distribution of the node degree
	*Circular Graph*		functional connectivity graph

$$ISI_{n}=Peak_{n}-Peak_{n-1}$$

where *Peak* is the time of peak of the *n*^*th*^ event. **Interpretation:** A greater ISI indicates slower Ca^2+^ activity.

***Note: AverageFrequency -***The average frequency of Ca^2+^ events of all ROIs (events/min). **Interpretation:** A greater average frequency indicates faster neuronal activity.

***Note: PercentageActiveROI -*** The percentage of ROIs with at least one recorded calcium event. It represents the fraction of the entire sample active during the measurement (Figure 6B – Green hexagon: Active ROI; Gray Hexagon: Inactive ROI). ***Interpretation:*** A greater value suggests a larger proportion of active surface area of the sample.

***Note: SyncIndex (Synchronization Index) -*** The global synchronization index of the sample. It quantifies the global degree of synchronization of multiple time series recorded simultaneously from multiple ROIs. It is important to notice that the entire traces and not the firing patterns are used to compute the synchronization index. **Interpretation:** A greater global synchronization index suggests that the sample activity is more synchronous (Figure 6B).

***Note: N-*** The number of ROIs/nodes. It is customized by the user while creating the ROI mask (ex. Figure 6 – seven ROIs).

***Note: NodeDegree (Node Degree) -*** The node degree (Figure 6C - the numbers refer to the central green ROI), normalized to the maximum number of edges (N-1), calculated as:

$$node\;degree=\frac{1}{N-1}\sum_{i=1}^{N}degree_{i}$$

In a weighted graph, $degree_{i}$ is the sum of the edge weights for edges directly connected to node_i_. In a binary graph, $degree_{i}$ is the number of edges directly connected to node_i_. ***Interpretation*:** A greater node degree suggests a stronger connectivity of the i^th^ node to the other nodes.

***Note: Node degree distribution -*** A Kernel density estimation graph showing probability distribution *versus.* node degree value. ***Interpretation***: A distribution curve with a long tail may suggest the existence of hub nodes – a small number of nodes with node degree greatly exceeding the average. Real world networks (i.e., brain, internet, air traffic network) tend to possess a long tail distribution, reflecting the presence of hub nodes (Bullmore and Sporns, 2009).

***Note: Modules and Modularity -*** A module is a group of nodes that are densely/strongly connected within modules and weakly/sparsely between nodes belonging to other modules (Figure 6D – green, red, and blue modules). Modularity is a value that measures the strength by which the network can be partitioned into modules. Number of modules and module assignment of nodes in a network are identified by maximizing the network’s modularity during computation. ***Interpretation*:** Real world networks tend to divide naturally into communities or modules. A greater modularity indicates stronger community structure of the network (Newman, 2006).

***Note: NumberofModules (Number of Modules) -*** The number of modules in which the network has been partitioned (Figure 6D – green, red, and blue modules).

***Note: ModulesComposition (Modules Composition) -*** A table summarizing the module index (first column), the number of ROIs in each module (second column) and the node indices in each module (third column).

***Note: AverageEdgeWeight (Average Edge Weight) -*** The average value of the normalized node degree of the sample. ***Interpretation*:** In a weighted graph, it represents the average edge weight of a node. In a binary graph, it represents the average probability that a node is connected to another one. Larger values correspond to greater average connectivity of the network.

***Note: AverageClustCoeff (Average Clustering Coefficient) -*** The average clustering coefficient of a network (Figure 6E) is calculated as the average of the local clustering coefficients *CC*_*i*_ of all the nodes as follows:

$$Average\;Clustering\;Coefficient=\frac{1}{N}\sum_{i=1}^{N}CC_{i}$$

In a weighted network, *CC*_*i*_ represents the strength of triangle networks (Figure 6E, left) formed by three neighboring nodes and is calculated as follows (Onnela et al., 2005):

$$CC_{i}=\;\frac{1}{k_{i}\left(k_{i}-1\right)}\sum_{j,k}\frac{\sqrt[{3}]{w_{ij}w_{jk}w_{ik}}}{\text{max}\left(w\right)}\;$$

In a binary graph, *CC*_*i*_ represents the probability that two nodes *j* and *k* are connected to each other while they are both connected to a node *i* (Figure 6E, right). ***Interpretation*:** A greater average clustering coefficient suggests stronger tendency of the network to form local clusters. Real world networks tend to have a high clustering coefficient (Muldoon et al., 2016).

***Note: AveragePathLength (Average Path Length) –*** The average path length of a network (Figure 6F) is calculated as:

$$Path\;Length=\frac{1}{N\left(N-1\right)}\sum_{i\neq j}d\left(i,j\right)$$

where *N* is the total number of nodes and *d(i,j)* is the shortest path between node *i* and *j* (Figure 6F – shortest path is indicated in red). For a weighted network, the shortest path is calculated as $d\left(i,j\right)=1/w_{ij}$, where $w_{ij}$ is the edge weight between the node *i* and *j*. For a binary network, the shortest path represents the smallest number of edges to connect node *i* and *j*. ***Interpretation*:** A greater average path length suggests that a longer distance or more nodes must be crossed to transmit the information between two random nodes. Average path length is also inversely correlated to the probability of shortcuts in the network. Real world networks tend to have a small value of the path length (Muldoon et al., 2016). It is also important to mention the debate on its interpretation in correlation-based functional networks. In short, paths in those networks represents sequences of statistical association but may not necessarily characterize the presence of information routes (Fornito et al., 2010).

### Quality control assays (optional)

The quality control assays provide additional procedures to examine the viability, development, and functionality of the neurons and 3D cultures. We recommend conducting the quality control assays at the same time as the experiments using the same batch of materials and cells, even though they are not required for the functional analysis.

### Quality control assay #1 – 2D

**Timing: Add 0.5–1 h to each day of the 3D culture steps**

This step describes conventional 2D culture of cortical neurons. We recommend conducting it using the same batch of neurons and for the same duration as in the 3D culture to ensure the viability of the neurons from the isolation procedure.

84. On day −1, coat tissue culture plates with PDL (0.1 mg/mL) and laminin (10 μg/mL) for 12–24 h.
85. On day 0, wash the coating with PBS three times (each with 5 min incubation) and once with Neuro Medium + 5% FBS (incubate until cell seeding).
86. Seed cells at 100–250k/cm^2^ in Neuro Medium + 5% FBS.
87. On day 1, remove all Neuro Medium + 5% FBS and replace with serum-free Neuro Medium.
88. Change 50% medium every 3–4 days.
89. Examine cell morphology frequently.

### Quality control assay #2 – drug response

**Timing: Add 15–30 min per sample on Ca**^**2+**^**imaging day**

This section describes using neurotransmitter receptor antagonists, namely bicuculline (GABA_A_ receptor blocker), picrotoxin (GABA_A_ and GABAρ receptor blocker), NBQX (AMPA receptor blocker), AP5 (NMDA receptor blocker), and tetrodotoxin (sodium channel blocker) on the 3D cultures to elicit known responses. We recommend conducting it after 2–3 weeks of culture and at the same time point as the control 3D cultures.

90. Dilute the 4 μL stock solution of bicuculline, picrotoxin, and NBQX with 76 μL Neuro Medium to make 100× working solution.
91. After acquiring a baseline fluorescence time-lapse, pipet 5 μL drug stock solution (100×) into the 500 μL medium. (Final concentrations: bicuculline 10 μM, picrotoxin 50 μM, NBQX 10 μM, AP5 25 μM, tetrodotoxin 20–40 μM).
92. Incubate for the following time: 10 min for picrotoxin and bicuculline, 20 min for NBQX, AP5, and tetrodotoxin.
93. Acquire post-treatment fluorescence time-lapse.

### Quality control assay #3 – viability staining

**Timing: 2 h**

This section describes viability staining of 3D cultures with Calcein-AM, which is taken up only by viable cells and becomes fluorescent in the green spectrum. This step can be done at any time point.

94. Add 500 μL Calcein-AM working solution to a well in a 48-well plate.
95. Transfer 3D culture into the well containing the Calcein-AM working solution. Incubate at 37°C for 30 min.
96. Aspirate the Calcein-AM working solution and wash once with PBS with 5 min incubation.
97. Image with green fluorescence filter.

### Quality control assay #4 – visualizing structural network

**Timing: Add 15 min to day 1 (AAV infection) and 2–3 h per sample to image on confocal microscope**

This section describes infecting the 3D culture with AAV to express RFP in the neurons, in order to visualize the structural neuronal network in the 3D culture using confocal microscopy.

98. Follow Cortical Neuron Isolation and Cell Seeding section to prepare 3D cultures. In the AAV Infection section, steps 31–33, infect the seeded scaffolds with AAV1-hSyn1-TurboRFP at 1–3 × 10^6^ GC/mL.
99. Between day 14 and 21, fix the 3D cultures with 4% paraformaldehyde/4% sucrose for 1 h followed by three 30-min PBS washes.
100. Acquire z-stack images on a confocal microscope (20× to 40× objectives recommended).

### Quality control assay #5 – gene expression

**Timing: Variable (5–6 h per 24 samples of RNA isolation, 3 h for cDNA synthesis, 3 h per 96-well plate of qRT-PCR)**

This section describes qRT-PCR analysis of gene expression of proteins associated with neuronal maturation and synaptic neurotransmission (See Key resources table and Table 3) of the 3D cultures shortly after cells seeding and as the neuronal networks develop.

**CRITICAL:** Use nuclease-free supplies. Keep mRNA samples on ice as much as possible.

101. Collect uninfected 3D cultures on day 1, 14, and 21 each in 600 μL RLT/βME buffer in a 1.5 mL nuclease-free microcentrifuge tube. Store at −80°C.Thaw samples on ice. Use a homogenizer pestle to manually grind 3D culture into fine pieces.
102. Use a motorized tissue grinder to further grind 3D culture for 20 s.

**Pause point:** Homogenized samples can be stored at −80°C.

***Note:*** Long-term storage at this step can significantly affect mRNA concentration. Perform mRNA isolation as soon as possible.

103. Transfer sample to a QIAshredder column and centrifuge at 21,000 × *g* for 2 min.
104. Isolate mRNA from the flow through using a Qiagen RNeasy Mini Kit according to the manufacturer’s protocol (https://www.qiagen.com/us/products/discovery-and-translational-research/dna-rna-purification/rna-purification/total-rna/rneasy-mini-kit/#resources) with the exception to elute the mRNA using only 22–24 μL of nuclease-free H_2_O.
105. Measure mRNA concentrations using a NanoDrop spectrophotometer.

**Pause point:** mRNA can be temporarily stored at −80°C, but it is recommended to reverse transcribe to cDNA as soon as possible.

106. Synthesize cDNA using Bio-Rad iScript gDNA clear cDNA synthesis kit according to the manufacturer’s protocol (https://www.bio-rad.com/en-us/product/iscript-gdna-clear-cdna-synthesis-kit?ID=NUU8WD15).

**Pause point:** cDNA can be stored long-term at −80°C.

107. Assume 100% reverse transcription efficiency (1 ng mRNA = 1 ng cDNA), dilute cDNA with nuclease-free water to 5 ng/μL.
108. Run quantitative real-time PCR (qRT-PCR) to detect mRNA expression using Taqman Gene Expression Assays according to the manufacturer’s protocol (http://tools.thermofisher.com/content/sfs/manuals/cms_041280.pdf). Each reaction contains 10 ng of cDNA (i.e., 2 μL), 7 μL nuclease-free water, 1 μL Taqman assay, and 10 μL Taqman Gene Expression Assay Master Mix.
109. Calculate relative mRNA expression fold change using Rn18s as the reference gene and normalizing to the day 1 sample.

**Table 3:** List of genes to detect in the 3D culture and their expected dCt values (normalized to 18s)

Gene	Encoded protein	Association	Day	dCt (Target gene – 18s)	
				Min	Max
*Dcx*	Doublecortin	immature neurons	1	9.57	13.97
			14	12.13	16.15
			21	13.58	18.27
*Pax6*	Paired Box Protein Pax-6	immature neurons	1	14.08	21.20
			14	17.06	23.34
			21	17.01	17.83
*Syn1*	Synapsin I	presynaptic terminal	1	14.39	19.00
			14	14.05	17.57
			21	12.59	17.84
*Shank3*	SH3/ankyrin domain gene 3	postsynaptic terminal	1	15.62	16.58
			14	15.37	15.73
			21	14.04	15.28
*Slc17a7*	Vesicular glutamate transporter 1	glutamate neurotransmission (presynaptic)	1	16.31	19.97
			14	13.96	16.22
			21	11.64	15.83
*Gria1*	Glutamate Ionotropic Receptor AMPA Type Subunit 1	glutamate neurotransmission (postsynaptic)	1	14.28	18.44
			14	14.32	17.13
			21	13.60	17.28
*Grin1*	Glutamate Ionotropic Receptor NMDA Type Subunit 1	glutamate neurotransmission (postsynaptic)	1	14.81	19.64
			14	14.27	18.14
			21	13.16	16.40
*Slc1a3*	Excitatory amino acid transporter 1	GABA neurotransmission (presynaptic)	1	14.27	18.95
			14	13.13	16.09
			21	12.12	16.90
*Gabra1*	Gamma-aminobutyric acid type A receptor alpha1 subunit	GABA neurotransmission (postsynaptic)	1	16.67	19.16
			14	14.62	16.46
			21	12.32	15.15

## Expected outcomes

### AAV infection and Ca^2+^ imaging

**Figure 7:**
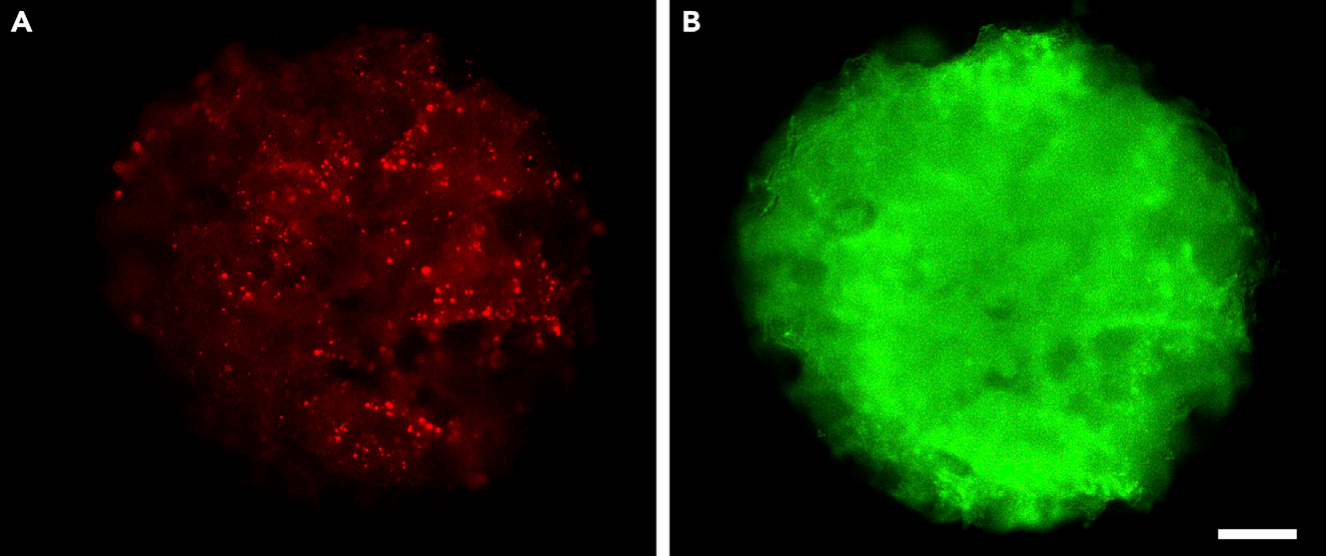
Fluorescence images of 3D culture (A) Nuclear dTomato and (B) GCaMP6f at 2 weeks of culture. Background fluorescence from silk scaffolds. Scale bar, 500 μm. See also Methods Video S10.

By two weeks, a large number of neurons in 3D cultures infected with AAV-hSyn1-GCaMP6f-P2A-nls-dTomato should express strong nuclear dTomato signal (Figure 7A). GCaMP6f has less prominent fluorescence signal (Figure 7B) and is not as clearly visible as dTomato. Spontaneous neuronal activities, observable as changes in GCaMP6f signal intensity, should be seen in clusters (> 30 μm) of neurons (See Methods Video S10). It should be noted that silk scaffolds are autofluorescent and do produce background in both the red and green channels. Non-homogenous distribution of cells is not unexpected.

Methods Video S10. Spontaneous neuronal activities in the 3D cortical culture at 3 weeks Time-lapse video of changes in GCaMP6f fluorescence intensity (related to the Expected outcomes section). Scaffold diameter = 3 mm. Video reused with permission from Dingle et al. (2020). See also Figures 4 and 7.

### Network activity and analysis

**Figure 8:**
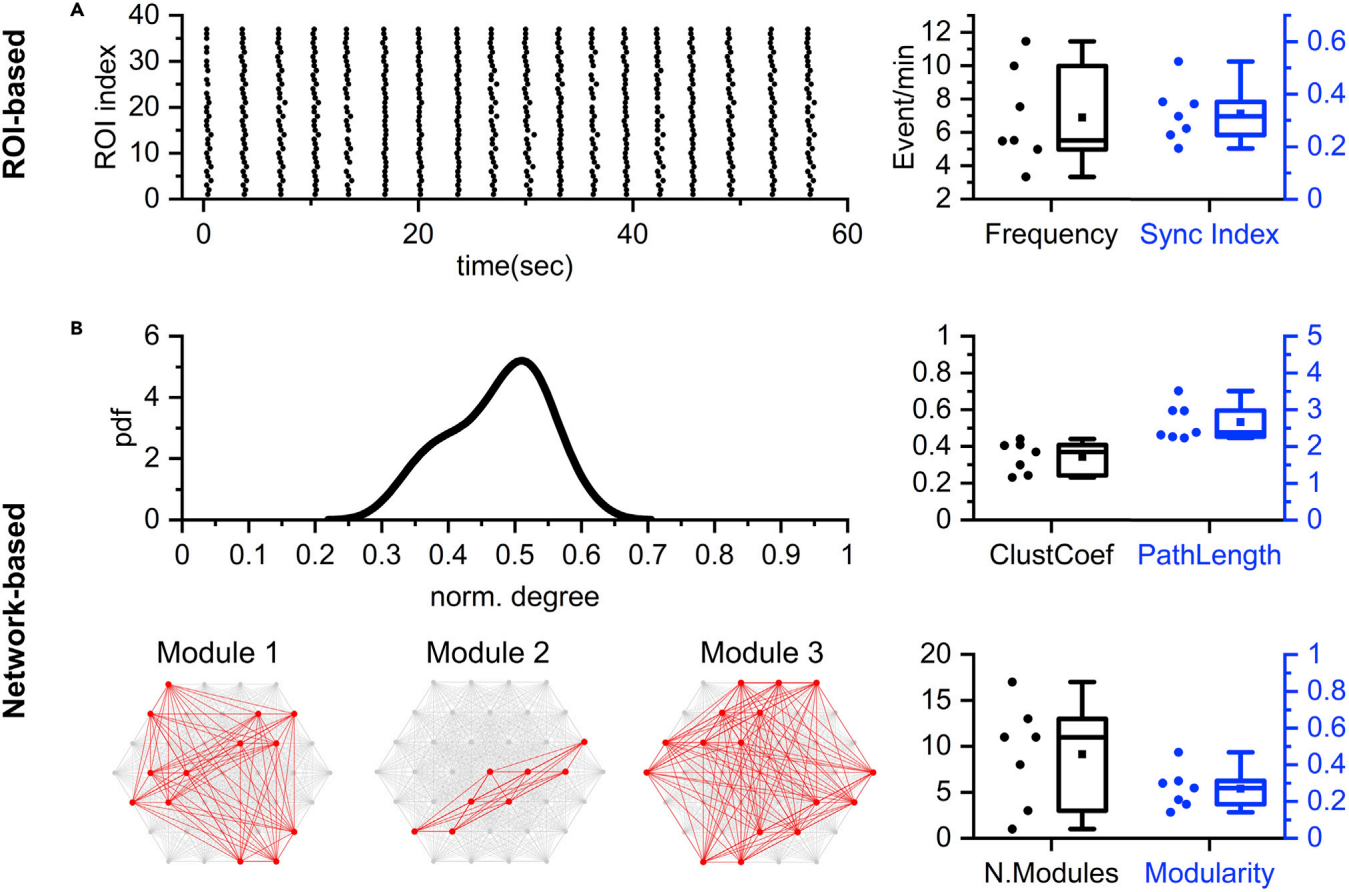
Examples of expected results of single ROI and network analyses (A) ROI-based analysis. Left: Raster plot of Ca^2+^ events per ROI versus time. Each dot represents a Ca^2+^ event. Right: Average Ca^2+^ event frequency and global synchronization index. Each dot represents a 3D culture sample. (B) Network-based analysis. Top left: Probability density function (pdf) versus normalized node degree of a representative culture. Top right: Average clustering coefficient and average path length. Bottom left: Module compositions of a representative 3D culture sample. Bottom right: Number of modules and modularity. Each dot in the scatter graphs represents a 3D culture sample. (A) and (B) All bar graphs are mean, first and third quartiles, and minimum and maximum. n = 7.

At 3 weeks, the 3D cultures are expected to show spontaneous and sustained activity in most of the regions (Figure 8A). Small clusters of neurons should have locally synchronized activities and Ca^2+^ transients should be visible under the microscope.

When FluoroSNNAP finishes, it should generate 6 files:

original_filename.xlsx
original_filename_summary.txt
analysis.mat
analysis-original_filename.mat
CaSignal-original_filename.mat
processed_analysis.mat

When *RunNetworkAnalysis.m* finishes running without errors, it should generate the following results in the workspace (additional items are also visible in the workspace). See Network analysis with RunNetworkAnalysis.m Section for descriptions of each variable

CalciumEventsTable
FinalTableNetworkData
ISITable
Matrix: This is the cross-correlation coefficient matrix extracted from the processed_analysis.mat
Modules
ModuleComposition
NodeDegree
SingleParameterTable: This contains AverageFrequency, PercentageActiveROI, and SyncIndex.

Two figures will be generated:

The first figure is Kernel Density Distribution of the node degree, with the average degree indicated as well.

The second figure is a circular graph showing the functional connectivity graph. For weighted graphs, the edge weight is directly proportional to the thickness of the lines.

In addition, an Excel file is automatically saved containing the outputs divided in different sheets (as detailed in the code comments section).

Example results from real samples are provided in Figure 8, using weighted network analysis.

A representative raster plot (each dot is the time of peak of a single calcium event) is shown in Figure 8A (left). The number of events/min and synchronization index are then graphed for 7 samples (Figure 8A - right). Upon network reconstruction (weighted network), the probability density function (pdf) of the normalized node degree is generated, as shown in the representative distribution of Figure 8B (top, left). The curve had a Gaussian-like profile suggesting the absence of hub nodes, namely nodes with a high node degree. Two topological descriptors, the average clustering coefficient and average path length, are computed from the network (Figure 8B – top, right). Finally, the network is partitioned into modules (Figure 8B – bottom, left; red nodes and edges) based on the computation of the modularity (Figure 8B – right, bottom).

### Quality controls

**Figure 9:**
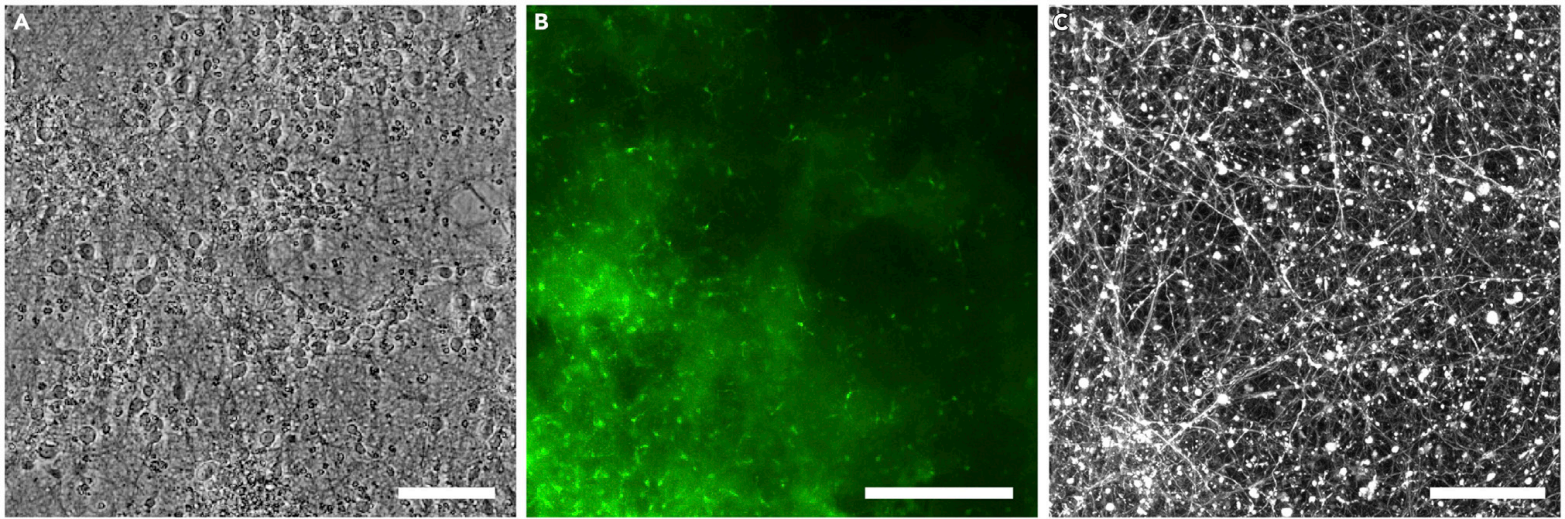
Representative images of expected results from quality control assays (A) Phase contrast image of 2D morphology of cortical neurons at 3 weeks. (B) Fluorescence image of live cells in 3D culture at 2 weeks, indicated by calcein (green). (C) Reconstructed confocal z-stack image of TurboRFP+ neurons in the 3D culture. Scale bars, (A) 100 μm (B) 200 μm (C) 50 μm.

QC #1 - In 2D cultures, short neurites emerged on day 1. Visual networks with increasing density form between day 3 and 7 (Figure 9A).

QC #2 - When 3D cultures are treated with picrotoxin or bicuculline, Ca2+ event frequency increases. When 3D cultures are treated with NBQX or AP5, Ca2+ event frequency decreases or ceases. When 3D cultures are treated with tetrodotoxin, all or almost all activities are eliminated.

QC #3 - High density of live calcein-positive cells throughout the 3D culture. 3D cultures are expected to have lower density of viable cells at 2–3 weeks of culture than at <1 week (Figure 9B).

QC #4 - In AAV-syn1-TurboRFP infected 3D cultures, strong TurboRFP expression in neurons at 2–3 weeks of culture is observed, demonstrating an extensive neuronal network with long neurites (hundreds of μm). When visualized using confocal microscopy with z-stack imaging, observations of many small and large round objects are expected (Figure 9C).

QC #5 - A 400–1,200 ng mRNA yield per 3D culture. Significant decreases of *Dcx* and *Pax6* and increases of *Syn1, Shank3, Slc17a7, Gria1, Grin1, Slc1a3,* and *Gabra1* from day 1 to day 14 and day 21. See Table 3 for dCt (Ct_target gene_ − Ct_18s_) value ranges obtained from real samples.

## Limitations

Network activity depends on the health of the primary neurons and the distribution of cells within the scaffold. Therefore, the cultures are prone to sample-to-sample, batch-to-batch, and/or user-to-user variability. The network analysis is based on the projection imaging of the 3D culture, and therefore the z-direction network information flow cannot be inferred from this protocol. Ultra-speed confocal microscopy, such as light sheet confocal microscopy, will be required to obtain x,y,z information at the speed necessary to detect Ca^2+^ transients. This protocol focuses on wide-field, mesoscale network analysis among populations of neurons. It relies on synchronized Ca^2+^ transients in local clusters of neurons for a Ca^2+^ event to be detectable per ROI. If the neuronal activity is mainly asynchronous, Ca^2+^ event detection becomes unreliable and cannot be distinguished from the background signal. When 3D cultures are under conditions where there will be low or no activity, global synchronization index may become artificially high due to the similarly of background noise, and network analysis cannot be reliably performed.

This protocol has the potential to be adapted to analyze neural networks of human iPSC-derived or neural stem cell-derived neurons. Key issues to consider include: 1) the AAV-GCaMP6f infection step needs to be optimized for human neuronal cultures and may require repeated AAV infections to maintain adequate GCaMP levels, 2) human neurons mature at a slower rate than rodent neurons and synapsin I expression is required for GCaMP6f expression, and 3) functional neural network formation may take longer in human cultures than in rodent cultures and therefore Ca^2+^ imaging time points may need to be adjusted accordingly.

## Troubleshooting

### Problem 1

Silk fibroin not fully dissolved after 4 h in 60°C (Before you begin step 12).

### Potential solution

Inaccurate LiBr solution concentration due to the stock LiBr powder absorbing too much moisture from air. Use new LiBr and keep the container closed and parafilm-sealed as much as possible.

### Problem 2

Silk solution became opaque and hardens (Before you begin step 20).

### Potential solution

Silk solution is in storage for too long and starts to form insoluble β-sheet structure. Prepare fresh silk solution and use it within 2 weeks.

### Problem 3

Sponge is not homogenous with visible large pores and cracks (Before you begin step 26).

### Potential solution

NaCl must be dry with individual particles well-separated and not in clumps. NaCl must be poured evenly when preparing the silk sponge. Practice pouring NaCl into an empty petri dish.

### Problem 4

Sponges/scaffolds appear fragile and are breaking apart (Before you begin step 27).

### Potential solution

Sponges/scaffolds are likely too dry at some point. Make new sponges and always keep them hydrated.

### Problem 5

No or faint GCaMP signal (major step 52).

### Potential solution

Check dTomato signal. Image the side with higher dTomato expression, which also indicates higher GCaMP expression. Perform Ca^2+^ imaging no earlier than 2 weeks. Use lowest fluorescence light output to reduce photobleaching. Make sure exposure time is not too long. Check the viability of the 3D cultures (See Quality Control #3). Check AAV quality and infection efficiency with 2D culture.

### Problem 6

No Ca^2+^ transients (major step 52).

### Potential solution

Incubate the samples on the stage at 37°C for a longer duration after placing on the stage and before imaging. Check the actual temperature of the culture on the stage to ensure that it is within the range of 36°C–38°C. Overheating can quickly and irreversibly damage the culture. Check multiple 3D cultures. Treat 3D cultures with picrotoxin or bicuculline to induce activity (See Quality Control #2).

### Problem 7

FluoroSNNAP closes immediately after opening (major step 68).

### Potential solution

Run as an administrator. In PC, right click the software, select Run as Administrator.

### Problem 8

How to choose the functional connectivity method (major step 75).

### Potential solution

Given a dataset, different functional connectivity approaches result in different networks. It is important to notice that there is no privileged technique. Each method comes with pros/cons that must be considered by the user, whom should choose based on the type of question to answer and the mathematical meaning of each approach. For further discussion, please refer to “Fundamentals of brain network analysis” (Fornito et al., 2016).

### Problem 9

FluoroSNNAP freezes at the end of functional analysis (major step 76).

### Potential solution

FluoroSNNAP can crash if there is little or no activity to perform network analysis. Check the original time-lapse images to visually determine whether there is enough activity. Run only the “Calcium Events Detection” module in FluoroSNNAP and check the Ca^2+^ peaks in the resulting .txt file. Restart the software and/or restart the computer.

### Problem 10

Error while running *RunNetworkAnalysis.m.* (major step 80).

### Potential solution

Make sure Bioinformatics Toolbox is installed. Check folder directory.

### Problem 11

How to choose a threshold for binarization (major step 82).

### Potential solution

The choice of the binarization threshold is a crucial step since it shapes the resulting network. We can use prior knowledges of the system to determine the noise level of the weight edges between two nodes, thus filtering out irrelevant connections. If unsure about the choice of a threshold, analyze the weighted matrix.

### Problem 12

Negative edges in the functional connectivity matrix. The computation of path length does not admit negative values. In addition, the *circularGraph.m* will not accept negative entries (major step 83).

### Potential solution

The meaning of negative edge values in the computation of the path length is under debate. The first option is to choose an alternative functional connectivity method to avoid negative edges. In addition, the user can rescale the functional matrix between zero and one. If the code detects negative edges, it will display a dialog box reporting how many there were; moreover, it automatically substitutes them with zero entries (to allow the program to run). Finally, we can take the absolute value of each edge. For a recent survey, please refer to (Hallquist and Hillary, 2019).

## Resource availability

### Lead contact

Further information and requests for resources and reagents should be directed to and will be fulfilled by the Lead Contact, David L. Kaplan (david.kaplan@tufts.edu).

### Materials availability

This study did not generate new unique reagents.

### Data and code availability

The MATLAB code RunNetworkAnalysis can be downloaded at https://github.com/mattiabonzanni/Integrated-Functional-Neuronal-Network-Analysis-of-3D-Silk-Collagen-Scaffold-based-Cortical-Culture
